# Community Assembly and Co-Occurrence Patterns of Microeukaryotes in Thermokarst Lakes of the Yellow River Source Area

**DOI:** 10.3390/microorganisms10020481

**Published:** 2022-02-21

**Authors:** Ze Ren, Kang Ma, Xuan Jia, Qing Wang, Cheng Zhang, Xia Li

**Affiliations:** 1 Research and Development Center for Watershed Environmental Eco-Engineering, Advanced Institute of Natural Sciences, Beijing Normal University, Zhuhai 519087, China; wq@bnu.edu.cn (Q.W.); zhangcheng@bnu.edu.cn (C.Z.); lixiabnu@bnu.edu.cn (X.L.); 2 School of Environment, Beijing Normal University, Beijing 100875, China; mk@mail.bnu.edu.cn; 3 College of Education for the Future, Beijing Normal University, Zhuhai 519087, China; 202011039306@mail.bnu.edu.cn; 4 School of Engineering Technology, Beijing Normal University, Zhuhai 519087, China

**Keywords:** microeukaryotes, deterministic process, stochastic process, co-occurrence, Qinghai–Tibet Plateau

## Abstract

Thermokarst lakes are important aquatic ecosystems in cold regions, experiencing several changes due to global warming. However, the fundamental assembly mechanisms of microeukaryotic communities in thermokarst lakes are unknown. In this study, we examined the assembly processes and co-occurrence networks of microeukaryotic communities in sediment and water of thermokarst lakes in the Yellow River Source Area. Sediment microeukaryotic communities had a significantly lower α-diversity but higher β-diversity than water microeukaryotic communities. pH, sediment organic carbon, and total phosphorus significantly affected taxonomic and phylogenetic diversity of sediment communities, while conductivity was a significant driver for water communities. Both sediment and water microeukaryotic communities were strongly governed by dispersal limitation. However, deterministic processes, especially homogenous selection, were more relevant in structuring microeukaryotic communities in water than those in sediment. Changes in total nitrogen and phosphorus in sediment could contribute to shift its microeukaryotic communities from homogeneous selection to stochastic processes. Co-occurrence networks showed that water microeukaryotic communities are more complex and interconnected but have lower modularity than sediment microeukaryotic communities. The water microeukaryotic network had more modules than the sediment microeukaryotic network. These modules were dominated by different taxonomic groups and associated to different environmental variables.

## 1. Introduction

Thermokarst lakes are shallow thaw lakes formed after the thawing of ice-rich permafrost and widely distributed in cold regions with high altitude or latitude [1,2]. In the Arctic and sub-Arctic regions, as well as the Qinghai–Tibet Plateau (QTP), thermokarst lakes are common landscape features [3,4,5]. Thermokarst lakes show huge differences in lake size ranging from a few square meters to hundreds of square kilometers [6,7,8]. Moreover, thermokarst lakes also have significantly different geomorphological, physicochemical, and biological characteristics, even at a very small spatial scale [5,9,10,11]. Permafrost degradation continuously drives the evolution of thermokarst lakes in terms of their formation, expansion, shrinkage, and, finally, disappearance [12,13,14]. Accelerating climate change expedites the evolution processes of thermokarst lakes, leading to tremendous changes in lake environments and ecosystem processes [8] and exerting pressure on the organisms living in them. However, the fundamental mechanisms of community assembly in thermokarst lakes are still unknown and are important to understanding ecosystem stability and the function of thermokarst lakes in a warming world.

Microeukaryotic communities are a versatile component in lake ecosystems and encompass enormous diversity [15,16,17]. Composed by algae, fungi, protozoa, and metazoa through complex interactions [18,19], microeukaryotic communities play important roles in structuring the food web of aquatic ecosystems, and their assembly mechanisms and environmental responses have long been of research interest in microbial ecology [20,21,22]. Microeukaryotic community structure vary across different temporal and spatial scales [17,21,23,24]. Understanding the assembly rules controlling community diversity and biogeography is central in microbial ecology [25,26]. Microbial community is simultaneously influenced by stochastic and deterministic processes with differential importance [25,26,27]. Deterministic processes emphasize the importance of niche-based mechanisms, such as environmental filtering and biotic interactions [26]. On the contrary, stochastic processes highlight neutral processes, such as unpredictable disturbances, ecological drift, and probabilistic dispersal [28,29,30]. However, it is challenging to characterize those processes in microbial community variations [29,31,32]. In addition, according to the topological features of the interactions between taxa, co-occurrence networks reveal community assembly rules [33,34] by disentangling interactions in the microbiome, delineating keystone taxa, and finding modular structures [35,36]. For thermokarst lakes, we lack an understanding on microbial community assembly mechanisms.

In general, sediment and water are different in various aspects but also have intimate interconnections [37,38]. In thermokarst lakes, the sediment–water interactions were inherent from the lake formation and intensified by thermokarst processes, microbial activities, and wind-induced mixing [39,40,41]. Our previous studies of thermokarst lakes showed that sediment and water harbor distinct bacterial communities in terms of taxonomic composition, beta diversity, and co-occurrence networks [11,42]. Thus, it is also interesting to reveal the differences of community assembly rules between sediment and water microeukaryotic communities.

As the “Third Pole” of the Earth, QTP is extremely sensitive to anthropogenic activities and global climate change [43,44]. In the Yellow River Source Area (YRSA), it is predicted that the mean annual temperature will increase 1.8–3.5 ℃ by 2080 [45]. The ongoing global warming has already accelerated permafrost degradation, resulting in extensive changes of thermokarst lakes [8,46]. In this study, we investigated microeukaryotes in 23 thermokarst lakes in the YRSA on the QTP using 18S rRNA gene sequencing. We aimed to (1) assess the relative contribution of stochastic and deterministic processes in shaping microeukaryotic communities in sediment and water in the thermokarst lakes, and (2) disentangle the co-occurrence patterns of these microeukaryotic communities. The results can provide useful information for a better understanding and response prediction of thermokarst lakes in a warming future.

## 2. Methods

### 2.1. Study Area, Field Sampling, and Chemical Analyses

This study was conducted in the Yellow River Source Area, located in the northeast of the QTP (Figure S1). This area has a cold and semiarid climate with an annual precipitation of 300–800 mm [47], an annual mean air temperature between −4 and 2 ℃ [47], and annual evaporation of 800–1200 mm [48]. The average elevation is about 4500 m [4]. Permafrost and thermokarst lakes are widely distributed in this area [4,49], with permafrost covering up to 80% of the land [4].

In early July 2020, 23 thermokarst lakes were sampled in this area. Information on the lakes and sampling process has been provided in our previous publication [11]. In each lake, both water samples and sediment samples were collected with three replicates (subsamples). Thus, a total of 23 water samples and 23 sediment samples were collected. In each lake, three 1 L water subsamples were collected at a depth of 0.3 to 0.5 m and filled in acid clean bottles. Water microbial samples were collected by filtering 200 mL water from each of the three 1 L subsamples, respectively. For each lake, three filters were combined into one composite sample and frozen in liquid nitrogen immediately in the field and stored at −80 ℃ in the lab until DNA extraction. The remaining water was used for chemical analyses in the lab. The conductivity and pH of the lake water were measured in situ using a multiparameter instrument (YSI ProPlus, Yellow Springs, OH, USA). For water samples, dissolved organic carbon (DOC), total nitrogen (TN), and total phosphorus (TP) were analyzed. DOC was analyzed on filtered (using pre-combusted GF/F filters) lake water using a Shimadzu TOC Analyzer (Shimadzu, Columbia, MD, USA). TN was measured by ion chromatography after persulfate oxidation (EPA 300.0). TP was measured using the ascorbic acid colorimetric method after persulfate oxidation (EPA 365.3). In each lake, sediment samples were collected using a Ponar Grab sampler from three points (subsamples). For each subsample, the top 5 cm of the sediment was collected and homogenized. For each lake, sediment microbial sample was collected by mixing three 15 mL subsamples in a 45 mL sterile centrifuge tube and freezing it in liquid nitrogen in the field. The remaining sediments were used for chemical analyses in the lab. For sediment samples, pH, conductivity, sediment organic carbon (SOC), TN, and TP were analyzed. SOC was measured by the potassium dichromate oxidation spectrophotometric method (Chinese standard method HJ615-2011). Sediment TN was measured using the modified Kjeldahl Method (Chinese standard method HJ717-2014). Sediment TP was measured using the ascorbic acid colorimetric method after microwave extraction with nitric acid [50]. Chemical properties of sediment and water samples were summarized in our previous study [11].

### 2.2. DNA Extraction, PCR, and Sequencing

DNA was extracted using the TIANGEN-DP336 DNA Isolation Kit (TIANGEN-Biotech, Beijing, China) following the manufacturer’s instructions. DNA extracts were quantified using a Qubit 3.0 Fluorometer (Life Technologies, Darmstadt, Germany). A total of 50–100 ng DNA was used to generate amplicons. The 18S rDNA hypervariable regions V4-V5 were amplified using forward primers 817F-5′-TTAGCATGGAATAATRRAATAGGA-3′ and reverse primer 1196R-5′-TCTGGACCTGGTGAAGTTTCC-3′. The PCR reaction was conducted on a thermal cycler (ABI GeneAmp^®^ 9700,Foster City, CA, USA) using the following program: 5 min initial denaturation at 94 °C, 30 s denaturation at 94 °C with 26 cycles, 30 s annealing at 56 °C, 20 s extension at 72 °C, and finally 5 min extension at 72 °C. DNA libraries were verified on 2% agarose gels (Biowest agarose, Madrid, Spain) and quantified using a Qubit 3.0 Fluorometer (Life Technologies, Germany). DNA libraries were multiplexed and loaded on an Illumina MiSeq platform according to the manufacturer’s instructions (Illumina, San Diego, CA, USA). Raw sequence data were analyzed using QIIME 1.9.0 [51]. Sequences were quality filtered and clustered to generate operational taxonomic units (OTUs) at a threshold of 97% similarity against the SILVA 132 database [52] using QIIME. Raw sequence data can be accessed at the China National Center for Bioinformation (PRJCA005279).

### 2.3. Analyses

A null model analysis was used to estimate assembly processes of microeukaryotic communities in sediment and water of the thermokarst lakes using the picante v1.8.2 package in R [53]. Turnover in phylogenetic composition between communities was quantified using beta mean nearest taxon distance (βMNTD). The influence of deterministic processes (heterogeneous selection and homogeneous selection) on community assembly was estimated by evaluating the difference between the observed βMNTD and the mean of the null distribution of βMNTD in units of standard deviation, which is the beta nearest taxon index (βNTI). βNTI values <−2 or >+2 indicate less than or greater than expected phylogenetic turnover, representing signals for heterogeneous selection and homogenous selection, respectively [25]. On the other hand, if −2 < βNTI < 2, compositional differences are explained by stochastic processes (dispersal limitations and homogenizing dispersal). To assess the relative influences of the processes not assigned to deterministic processes, a Raup–Crick metric of taxonomic β-diversity (RC_Bray_) was used to analyze pairwise comparisons of the observed and expected taxonomic turnover (at OTU level) between communities. The fraction of pairwise comparisons with −2 < βNTI < 2 and RC_Bray_ < −0.95 were attributed to homogeneous dispersal, while those with −2 < βNTI < 2 and RC_Bray_ > 0.95 were attributed to dispersal limitation. Pairwise comparisons falling within null distribution of both metrics of phylogenetic and taxonomic β-diversity (−2 < βNTI < 2 and −0.95 < RC_Bray_ < 0.95) were assigned to “undominated”, representing the fraction that was not strongly governed by any single process [25,26].

Co-occurrence networks of sediment and water microeukaryotic communities were constructed by pairwise correlations (Spearman correlation) between OTUs. OTUs with an average relative abundance > 0.01% and present in more than 6 samples were used. *p*-values of Spearman correlation were corrected using the FDR method [54]. Only strong (Spearman’s R > 0.6 or R < −0.6) and significant (*p* < 0.01) correlations were used in network construction. Meanwhile, random networks (permutation = 999) with the same number of nodes and edges as the community networks investigated in this study were generated using the Erdos–Renyi model with the igraph 1.2.6 package [55]. Topological parameters such as average degree (average number of neighbors for all nodes, explaining complex pairwise connection), clustering coefficient (a measure of the local connectivity associating to network robustness), average path length (the average shortest path lengths connecting the node to all other nodes), and graph density as well as modular structure of the networks were analyzed using the igraph package. Differences of topological parameters between the sediment microeukaryotic network (SMN) and the water microeukaryotic network (WMN) were assessed using *t*-test (different low case letters indicate a significant difference of *p* < 0.05).

α-diversity indices, including Chao 1, observed OTUs, Shannon, and phylogenetic diversity (PD whole tree) were calculated using QIIME 1.9.1 [51]. β-diversity in terms of taxonomic and phylogenetic turnover was assessed using Bray–Curtis distance (based on the relative abundance of OTUs) and βMNTD, respectively. To estimate the habitat niche occupied by each species, we calculated the Levins niche width [56] using the “spaa” package [57] in R. The formula is $B_{i}=1/\sum_{1}^{n}p_{i}^{2}$. *B_i_* represents the niche width of OTU_i_ across the communities, *n* is the total number of communities, and *p_i_* is the proportion of OTU_i_ in each community. Species with a higher niche width are distributed more evenly along a wider habitat range than those with a lower niche width. Differences of α-diversity and β-diversity between sediment and water samples were assessed using a Wilcoxon rank-sum test. Principal coordinates analysis (PCoA) was performed to assess differences of microeukaryotic communities between sediment and water samples based on Bray–Curtis distance and βMNTD. Mantel tests were conducted to test the relationships between environmental variables and microeukaryotic community properties, including βNTI, βMNTD, Bray–Curtis distance, major network modules (modules with more than 30 nodes), and major taxonomic groups. All statistical analyses were carried out in R 4.0.5 [58].

## 3. Results

### 3.1. General Patterns of Microeukaryotic Communities in Sediment and Water

**Figure 1:**
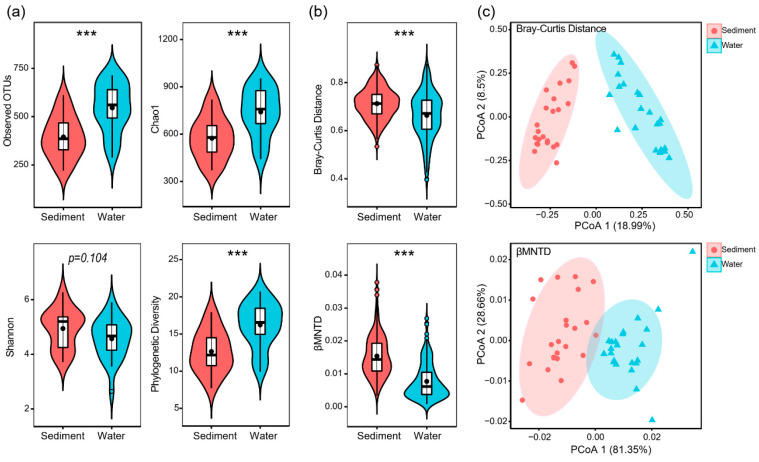
Distinct patterns of microeukaryotic communities in sediment and water samples. (**a**) Alpha diversity. (**b**) Beta-diversity. The differences of diversity indexes between sediment and water samples were tested using Wilcoxon rank-sum test (*** *p* < 0.001). (**c**) Principal coordinates analysis (PCoA) based on Bray–Curtis distance and βMNTD.

After quality filtering, a total of 2,032,004 high-quality sequences were obtained and clustered into 3141 OTUs. However, only 6 OTUs were found in all samples (Figure S2). Alpha diversity indexes (observed OTUs, Chao1, and phylogenetic diversity) were significantly lower in sediment samples than in water samples, except for the Shannon Index (Figure 1a). However, taxonomic β-diversity (shown as the Bray–Curtis distance) and phylogenetic β-diversity (shown as βMNTD) were significantly higher in sediment samples than in water samples (Figure 1b).

**Figure 2:**
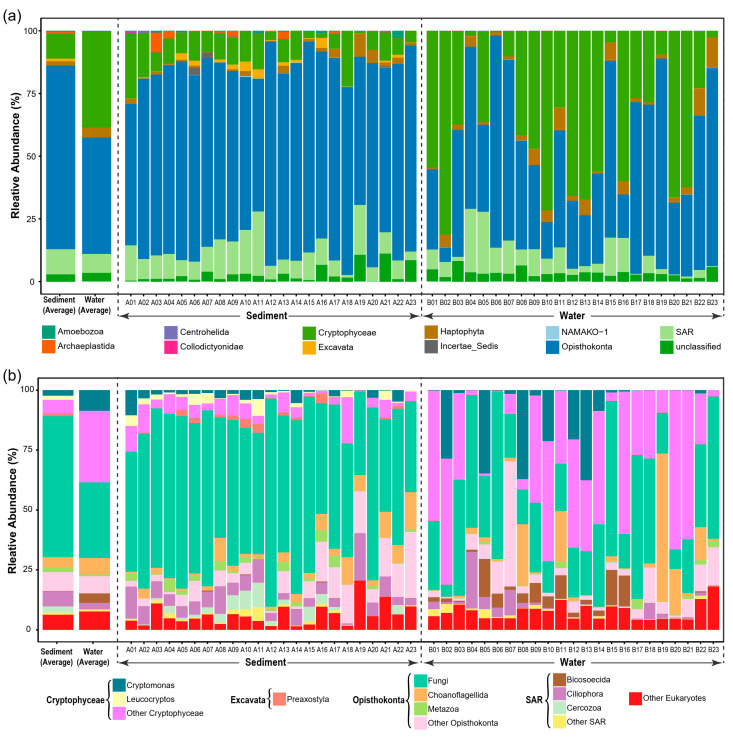
Taxonomic composition of the microeukaryotic communities in sediment and water samples. (**a**) Relative abundance of the super groups. (**b**) Relative abundance of the major lineages of the super groups.

Microeukaryotic communities also showed a distinct composition between sediment and water samples. Principal coordinates analysis (PCoA) based on Bray–Curtis distance and βMNTD indicated that microeukaryotic community composition was significantly different between sediment and water samples (Figure 1c). The phylogenetic diversity of microeukaryotic OTUs covered 11 supergroups (Figure 2a). Opisthokonta, Cryptophyceae, and SAR (Stramenopiles–Alveolata–Rhizaria) were dominant (mean relative abundance >5%) in both sediment and water samples (Figure 2a). In sediment samples, Fungi and Ciliophora were the dominant lineages with a mean relative abundance of 58.9% and 6.4%, respectively (Figure 2b). In water samples, however, Fungi, *Cryptomonas* sp., and Choanoflagellida were the dominant lineages with a mean relative abundance of 31.5%, 8.6%, and 6.9%, respectively (Figure 2b).

To identify physicochemical properties that affect dissimilarities of microeukaryotic communities and major lineages, we correlated these values to the differences in physicochemical properties between each pairwise set of samples (Table 1 and Table 2). For sediment samples, pH, SOC, and TP were strong and positive predictors for dissimilarities of microeukaryotic communities as well as many major lineages (Table 1). For water samples, however, conductivity and DOC were strong predictors for community dissimilarities and many major lineages (Table 2).

### 3.2. Assembly Processes

**Figure 3:**
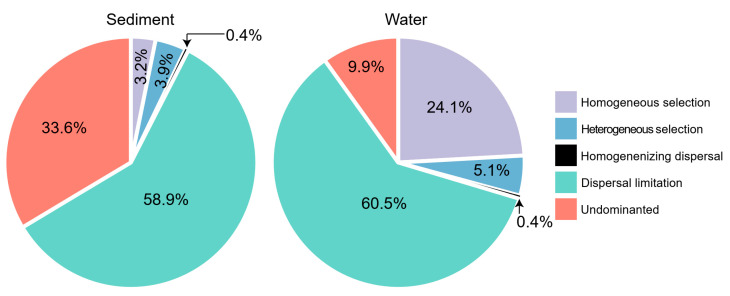
The contribution of deterministic (homogeneous and heterogeneous selection) and stochastic (dispersal limitations and homogenizing dispersal) processes to the turnover in the assembly of sediment and water microeukaryotic communities in thermokarst lakes. “Undominated” represents the fraction that was not dominated by any single process.

The quantification of phylogenetic turnover showed that stochastic processes (homogenizing dispersal and dispersal limitation) was dominant in both sediment (59.3%) and water (60.9%) microeukaryotic communities (Figure 3). Dispersal limitation contributed the most in the stochastic assembly. Deterministic assembly (homogeneous selection and heterogeneous selection) was much higher in water (29.2%) microeukaryotic communities than in sediment (7.1%) microeukaryotic communities (Figure 3).

The relationships between βNTI and major environmental variables were used to estimate changes in the relative influences of deterministic and stochastic assembly processes. Mantel tests showed that TN and TP were the best predictors of assembly processes for sediment microeukaryotic communities (Table 1). Pairwise βNTI values for sediment microeukaryotic communities were significantly and positively correlated with differences in TN and TP (Table 1), suggesting that an increasing divergence of TN and TP could contribute to a shift from homogeneous selection to stochastic assembly and finally to heterogeneous selection in the assembly of sediment microeukaryotic communities. However, none of the tested environmental variables had significant relationships with βNTI of water microeukaryotic communities (Table 2).

### 3.3. Co-Occurrence Networks

**Figure 4:**
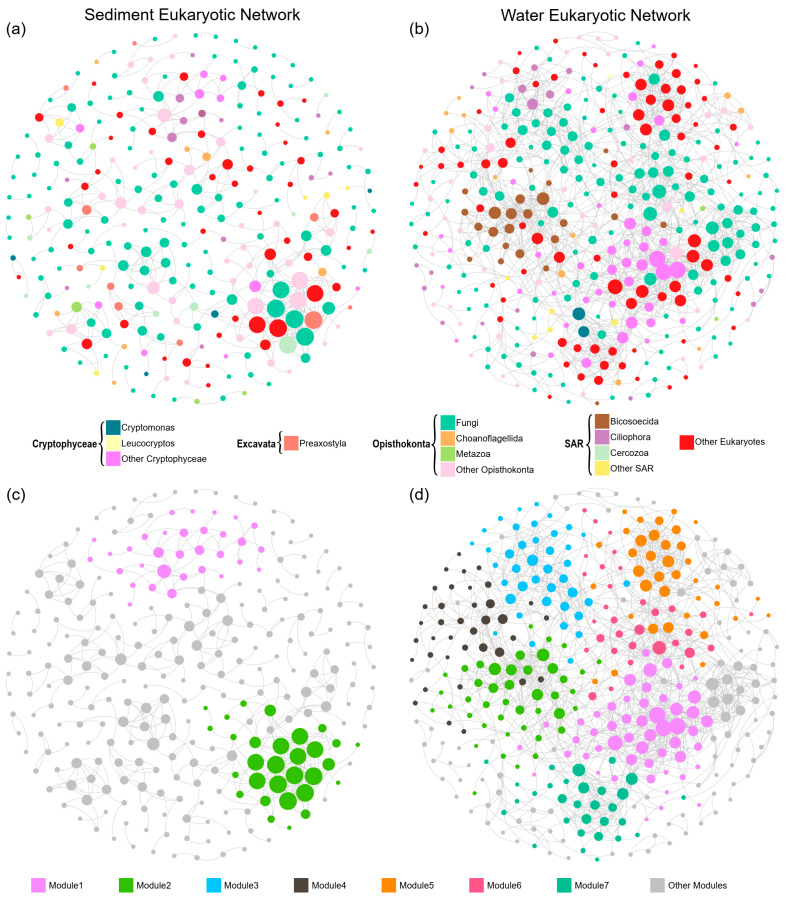
Co-occurrence networks of sediment and water microeukaryotic communities colored by (**a**,**b**) major taxonomic groups and (**c**,**d**) major modules (modules have more than 30 nodes). Nodes represent OTUs. Edges represent Spearman’s correlations. Only strong (Spearman’s R > 0.6 or R < −0.6) and significant (*p* < 0.05, *p*-values are adjusted using FDR methods) correlations are shown. The node size is proportional to the degree of the node.

**Figure 5:**
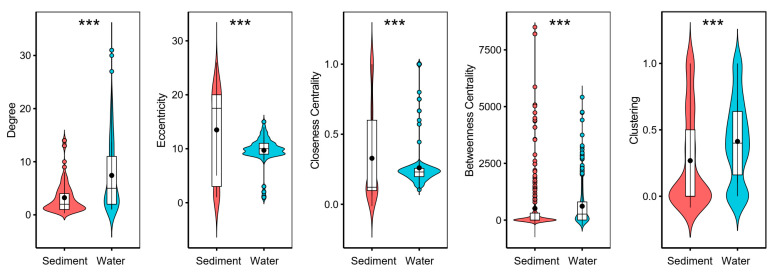
Node-level topological features of sediment and water microeukaryotic networks. The differences were tested using Wilcoxon rank-sum test (*** *p* < 0.001).

We generated co-occurrence networks for sediment and water microeukaryotic communities based on pairwise correlations. The sediment microeukaryotic network (SMN) was composed by 284 OTUs with 458 associations (Figure 4 and Table 3). The water microeukaryotic network (WMN) was composed by 376 OTUs with 1395 associations (Figure 4 and Table 3). A set of network-level and node-level topological features were calculated (Figure 5 and Table 3). The average degree, clustering coefficient, and graph density were higher in WMN than SMN, suggesting that OTUs in WMN were more interconnected. Moreover, the average path length and diameter were lower in WMN than in SMN, revealing closer relationships among water microeukaryotic communities. These results indicated that OTUs in water microeukaryotic communities co-occurred more frequently than that in sediment microeukaryotic communities. For the node-level topological features, the average values of eccentricity and closeness centrality were significantly higher, while degree, betweenness centrality, and clustering were significantly lower in SMN OTUs than in WMN OTUs (Figure 5).

**Figure 6:**
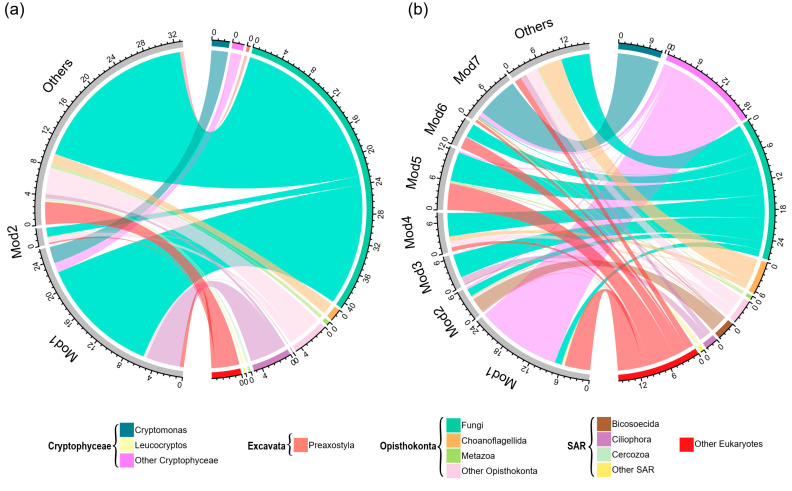
Taxonomic composition of modules in terms of relative abundance of OTUs in (**a**) sediment microeukaryotic network and (**b**) water microeukaryotic network.

In addition, both SMN and WMN had a significant modular structure (Figure 4). SMN had a significantly higher modularity but a smaller number of nodes than WMN, suggesting that SMN was composed by more small modules than WMN (Figure 4). SMN only had 2 major modules (module with more than 30 nodes), while WMN had 7 major modules (Figure 4). These modules had different taxonomic compositions (Figure 6). A Mantel test showed that these major modules responded differently to environmental properties (Table 1 and Table 2). For example, in SMN, module1 had strong correlations with pH, SOC, and TP, while module2 did not correlate with these environmental variables (Table 1). In WMN, module2 had strong correlations with all these environmental variables while module5 did not correlate with any (Table 2).

## 4. Discussion

This study showed that microeukaryotic communities were significantly different in composition and structure between sediment and water in our studied thermokarst lakes (Figure 1c). Sediment microeukaryotic communities had significantly lower α-diversity but higher β-diversity than water microeukaryotic communities (Figure 1). This pattern was consistent with our previous study of bacterial communities in the same lakes [11]. In lake ecosystems, distinct microorganisms inhabit in sediment and water [59,60,61,62]. Microeukaryotic communities were composed by various taxa and structured differently in sediment and water (Figure 2). A significantly higher mean value of βMNTD for SMC indicates that microeukaryotes in sediment are less closely phylogenetically clustered than that in water [63]. Mantel test showed that pH, SOC, and TP were significantly associated to dissimilarities of microeukaryotic communities for sediment samples (Table 1), while conductivity was significant for water samples (Table 2). The environmental responses of microeukaryotic communities were different to bacterial communities which are more associated to nutrient factors [11].

Microeukaryotic community structure varies across different spatiotemporal scales and habitats [17,21,23,24]. Sediment and water harbor significantly different microbial communities [42,64]. Results of this study suggest that both sediment and water microeukaryotic communities are strongly governed by stochastic assembly processes, especially dispersal limitation (Figure 3). Microbial dispersal is typically considered as a passive process [27] where increasing community variations and turnover are coupled with environmental filtering [65,66]. A strong signal of dispersal limitation and a very low signal of homogenizing dispersal indicate that movements of microeukaryotes between thermokarst lakes are highly restricted. A potential explanation for the high dispersal limitation is that thermokarst lakes are unique habitats and lack of connections due to their endorheic nature, resulting in strong simultaneous environment filtering. Moreover, thermokarst lakes are frozen for prolonged periods within a year, rendering microorganisms frozen in place as well [67,68]. Although many microorganisms have a cosmopolitan distribution, their slow dispersal may allow for regional phylogenetic differences and endemic taxa to develop in isolated habitats, resulting in low probabilities for microorganisms to disperse to suitable distant sites [69]. Therefore, dispersal processes may be restricted by limited movement, short unfrozen time, and strong environmental filtering, leading to the high dispersal limitation observed in thermokarst lakes, even at a small regional scale. This result is supported by other studies showing the significant effect of dispersal limitation on the structuring of microbial communities in lakes [70,71,72]. A similar pattern of strong dispersal limitation is also found for bacterial communities in permafrost, whose thawing results in the formation of thermokarst lakes [73]. In addition, microeukaryotic communities in water were more governed by deterministic assembly processes, especially by homogenous selection, than those in sediment (Figure 3). Long-term evolution of the thermokarst lakes results in homogenized habitats and homogeneous selection was found to be a stronger driver of the assembly of WMC than SMC. In addition, stronger governance by deterministic processes also suggests that the microeukaryotic communities in water were more phylogenetically clustered than that in sediment, supported by the lower Bray–Curtis distance and βMNTD of WMC than SWC (Figure 1).

Microeukaryotes typically co-occur with strong interactions in lake ecosystems [22,74,75], offering new insights into community assembly mechanisms by identifying potential interactions among microorganisms [33,76]. In our study, microeukaryotic communities in sediment and water constructed distinct co-occurrence networks in the studied thermokarst lakes. The results showed that the sediment and water microeukaryotic networks presented significantly different topological properties (Figure 4 and Figure 5, and Table 3). The network-level properties of WMN, such as higher number of nodes and edges, average degree, clustering coefficient, and graph density, as well as lower average path, indicate that WMN was more complex and more interconnected than SMN. A similar pattern was also found for bacterial community networks in these lakes [42]. Moreover, microeukaryotic communities had a higher α-diversity and lower β-diversity in water than in sediment, which is in accordance with the high complexity and interconnection of WMN. Higher taxa richness provides more probabilities for establishing interrelationships between each other [77]. Taxa also tend to co-occur at lower β-diversity because of high community similarity [42]. In addition, microeukaryotes had a higher niche width in water than in sediment (Figure S3), indicating that water microeukaryotes had stronger competition due to similar environmental preferences than sediment taxa. Species that have high competition or driven by the same environmental factors tend to have complex interactions with each other [78,79], resulting in a complex co-occurrence network. However, compared to SMN, WMN might have a lower stability under disturbance due to its strong connectivity and high complexity [80,81,82], thus suggesting a higher vulnerability of WMN in the accelerated warming world.

More ecological information on microeukaryotic communities, such as synergistic and competitive interactions as well as niche differentiation [83,84] can be uncovered by module structure of the networks [85,86]. SMN had a significantly higher modularity (the tendency of the network to contain modules) than WMN (Table 3), suggesting high habitat heterogeneity and niche diversity [62,87]. Moreover, WMN had a higher number of major modules (modules with more than 30 nodes) than SMN (Figure 4), suggesting that more taxa prefer similar environments and are functionally complementary [83,86]. Modules were dominated by different taxonomic groups (Figure 6). For SMN, the Mantel test showed that module1 was significantly associated with pH, SOC, and TP, while module2 was not significantly associated with any of the tested environmental variables (Table 1). For WMN, different modules responded differently to the environmental variables with module2 responding to all measured environmental variables and module5 responding to none (Table 2). The relationships between microbial modules and environmental variables provide further understanding of environmental influences on microbial assemblages [88,89].

## 5. Conclusions

Thermokarst lakes are pervasive and important aquatic ecosystems in cold regions with high latitude and elevation, experiencing significant changes due to accelerated global warming. Assessing the assembly mechanisms and co-occurrence patterns of microeukaryotic communities in sediment and water is critical for understanding the structure and stability of thermokarst lake ecosystems in the warming world. Our study highlighted that microeukaryotic communities in sediment and water have distinct alpha diversity patterns, assembly mechanisms, co-occurrence patterns, and different responses to environmental variables. Microeukaryotes in sediment were less phylogenetically and taxonomically clustered than those in water. SOC, pH, and TP were significant drivers of phylogenetic dissimilarities of microeukaryotic communities in sediment, while conductivity was significant driver for water microeukaryotic communities. Dispersal limitation was dominant in shaping microeukaryotic communities in both sediment and water. Moreover, deterministic assembly processes had a higher contribution to community assembly in water than that in sediment. Future changes in sediment TN and TP could shift assembly processes of sediment microeukaryotic communities. Water microeukaryotes are characterized by a highly complex and interconnected co-occurrence network with lower modularity than that of sediment microeukaryotes. It is expected that future climate change and permafrost degradation will impose different influences on sediment and water microeukaryotic communities in thermokarst lakes.

## Figures and Tables

**Table 1:** Mantel test between environmental variables and sediment microeukaryotic communities. Significant correlations (*p* < 0.05) were shown in bold with * and ** represent *p* < 0.05 and *p* < 0.01, respectively. NA represents not applicable.

	pH	Conductivity	SOC	TN	TP
βNTI	−0.041	0.075	0.115	0.175 **	0.137 *
βMNTD	0.197 **	0.084	0.145 *	0.116	0.146 *
Bray–Curtis Distance	0.250 **	−0.026	0.173 **	0.052	0.248 **
Module1	0.130 *	0.040	0.255 **	0.107	0.358 **
Module2	−0.016	0.067	−0.079	−0.035	0.071
*Cryptomonas* sp.	−0.015	0.074	0.223 **	0.131 *	0.236 **
*Leucocryptos* sp.	0.138 *	−0.112	−0.051	0.006	0.068
Preaxostyla	−0.069	0.208 **	−0.078	−0.028	−0.088
Fungi	0.190 **	−0.039	0.152 *	0.058	0.233 **
Choanoflagellida	0.043	0.039	−0.119	−0.091	0.004
Metazoa	0.198 **	0.187 **	0.056	0.069	0.127 *
Bicosoecida	NA	NA	NA	NA	NA
Ciliophora	0.122	−0.03	0.188 **	0.029	0.228 **
Cercozoa	0.172 **	0.165 **	0.037	0.098	0.281 **

**Table 2:** Mantel test between environmental variables and water microeukaryotic communities. Significant correlations (*p* < 0.05) were shown in bold with * and ** represent *p* < 0.05 and *p* < 0.01, respectively. NA represents not applicable.

	pH	Conductivity	DOC	TN	TP
βNTI	−0.023	0.011	−0.116	0.023	0.023
βMNTD	−0.041	0.134 *	0.067	−0.007	−0.004
Bray–Curtis Distance	0.071	0.144 *	0.183 **	−0.031	−0.034
Module1	0.118	0.063	0.129 *	−0.104	−0.091
Module2	0.126 *	0.480 **	0.342 **	0.155 *	0.267 **
Module3	−0.153 *	−0.043	0.021	−0.113	0.112
Module4	−0.001	0.321 **	0.105	0.065	0.208 **
Module5	0.082	0.032	−0.044	0.048	−0.063
Module6	0.192 **	0.011	−0.015	0.122	0.142 *
Module7	0.052	0.178 **	0.142 *	−0.042	−0.005
*Cryptomonas* sp.	0.008	0.156 *	0.133 *	−0.034	0.004
*Leucocryptos* sp.	−0.029	0.373 **	0.170 *	−0.057	−0.021
Preaxostyla	NA	NA	NA	NA	NA
Fungi	0.089	0.085	0.079	0.019	0.125 *
Choanoflagellida	0.112	0.097	0.101	0.406 **	0.203 **
Metazoa	0.032	0.115	0.038	0.006	0.023
Bicosoecida	0.092	0.458 **	0.347 **	0.040	0.332 **
Ciliophora	−0.155 *	0.045	−0.011	−0.144 *	0.121
Cercozoa	0.004	−0.030	−0.032	0.071	−0.088

**Table 3:** Comparison of topological parameters of co-occurrence networks investigated in this study (sediment microeukaryotic network and water microeukaryotic network) and their associated random networks (permutation = 999, values shown mean ± SD). The differences between sediment and water microeukaryotic network were assessed using *t*-test (different low case letters indicate the significant difference of *p* < 0.05).

Topological Parameters	Sediment		Water	
	This Study	Random	This Study	Random
Number of Nodes	284	284	376	376
Number of Edges	458	458	1395	1395
Negative Edges	12	12	140	140
Average Degree	3.225	3.225	7.420	7.420
Graph Density	0.011	0.011	0.02	0.02
Average Path Length	9.627 ^a^	4.821 ± 0.078	4.730 ^b^	3.177 ± 0.007
Diameter	26 ^a^	10.7 ± 0.879	15 ^b^	5.8 ± 0.413
Clustering Coefficient	0.460 ^a^	0.012 ± 0.005	0.528 ^b^	0.020 ± 0.002
Centralization Degree	0.038 ^a^	0.021 ± 0.003	0.063 ^b^	0.024 ± 0.004
Centralization Betweenness	0.201 ^a^	0.063 ± 0.014	0.069 ^b^	0.020 ± 0.004
Centralization Closeness	0.004 ^a^	0.013 ± 0.004	0.008 ^b^	0.097 ± 0.020
Modularity	0.836 ^a^	0.573 ± 0.01	0.639 ^b^	0.334 ± 0.006

## Data Availability

Raw sequence data can be accessed at the China National Center for Bioinformation (PRJCA005279).

## Supplementary Materials

The following supporting information can be downloaded at: www.mdpi.com/article/10.3390/microorganisms10020481/s1, Figure S1: Water and sediment samples were collected from 23 lakes in early July 2020 in the Yellow River Source Area on the Qinghai-Tibet Plateau. The map was cited from our previous research [11]; Figure S2: Flower plot diagram showing core and accessory OTUs across (a) all sediment and water samples, (b) all sediment samples, and (c) all water samples. The central circle shows the number of OTUs common to all samples while the petals show the number of OTUs in addition to the core set; Figure S3: Niche width of the taxa microeukaryotic communities of sediment and water. The difference was tested using Wilcoxon rank-sum test.
