# Supplementary material for: Community Assembly and Co-Occurrence Patterns of Microeukaryotes in Thermokarst Lakes of the Yellow River Source Area
Source: Microorganisms. 2022 Feb 21;10(2):481. doi: 10.3390/microorganisms10020481 (PMC8877526; doi:10.3390/microorganisms10020481)
Supplement: Supplementary file 1 [file microorganisms-10-00481-s001.zip › microorganisms-1591318-supplementary.pdf]

Supplementary Information

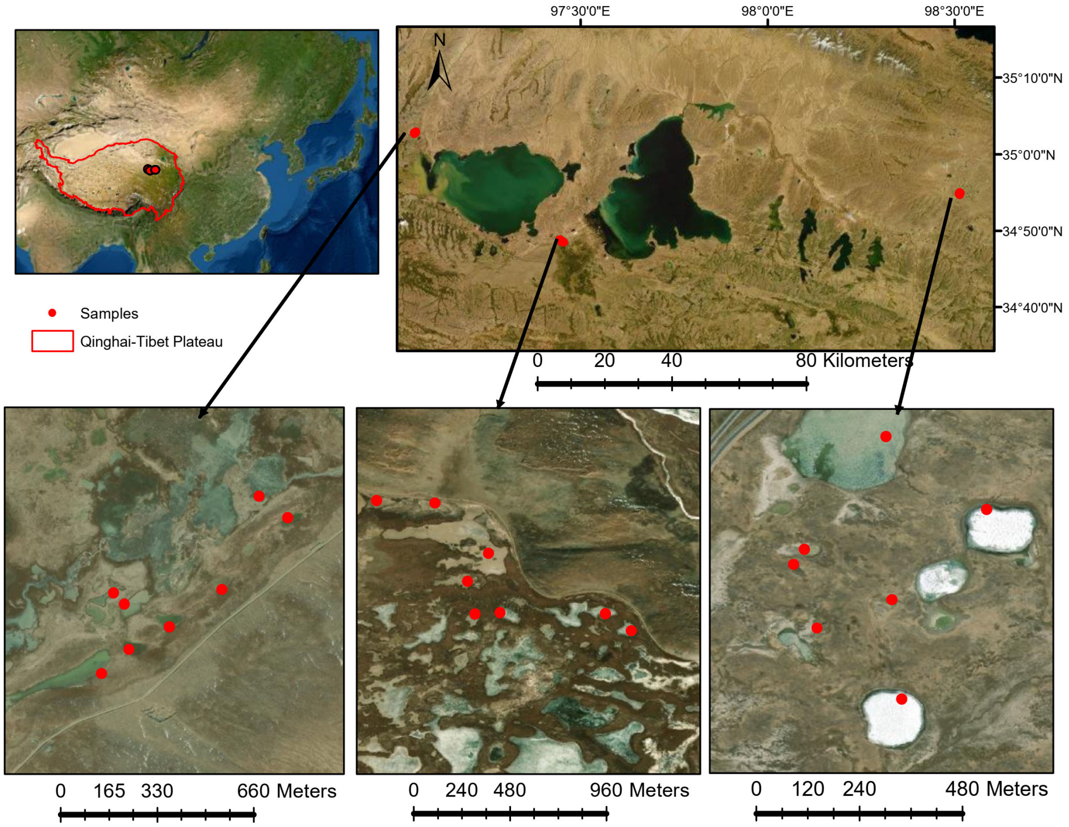

Figure S1. Water and sediment samples were collected from 23 lakes in early July 2020 in the Yellow River Source Area on the Qinghai-Tibet Plateau. The map was cited from Ren et al., 2021a.

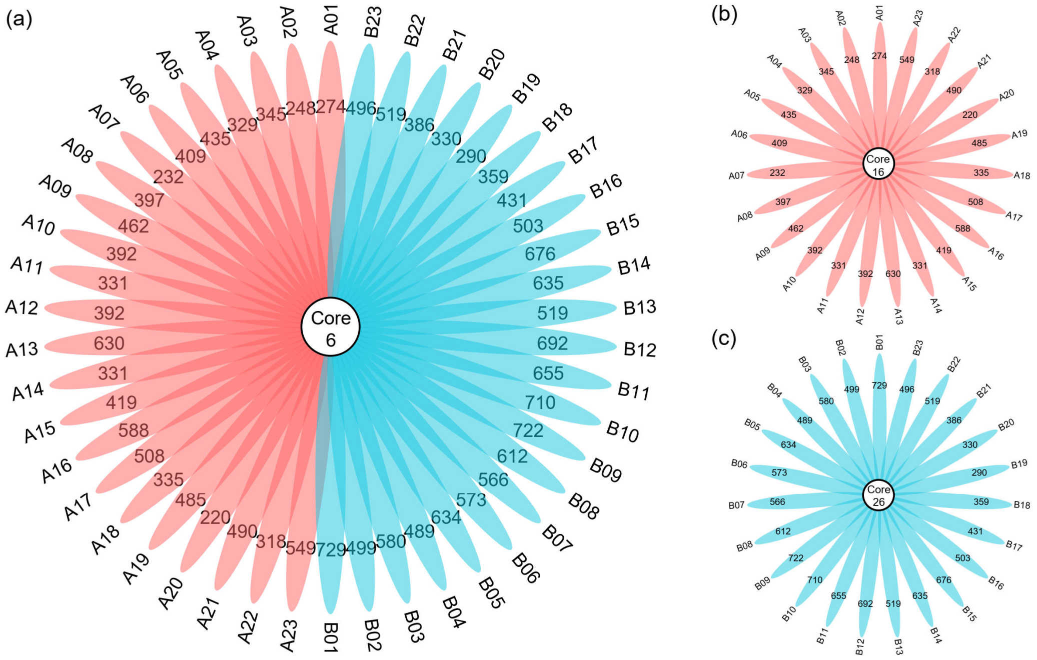

Figure S2. Flower plot diagram showing core and accessory OTUs across (a) all sediment and water samples, (b) all sediment samples, and (c) all water samples. The central circle shows the number of OTUs common to all samples while the petals show the number of OTUs in addition to the core set.

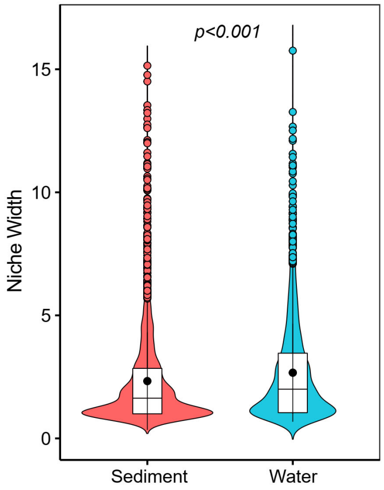

Figure S3. Niche width of the taxa microeukaryotic communities of sediment and water. The difference was tested using Wilcoxon rank-sum test.
